# Robustness of the BYM model in absence of spatial variation in the residuals

**DOI:** 10.1186/1476-072X-6-39

**Published:** 2007-09-20

**Authors:** Aurélien Latouche, Chantal Guihenneuc-Jouyaux, Claire Girard, Denis Hémon

**Affiliations:** 1 Inserm, U754, Villejuif, F-94807, France; 2 Univ Paris-Sud, IFR69, Villejuif, F-94807, France; 3 Univ Paris 5, Paris, F-75006, France; 4 CNRS, UMR8145, Paris, F-75006, France

## Abstract

**Background:**

In the context of ecological studies, the Bayesian hierarchical Poisson model is of prime interest when studying the association between environmental exposure and rare diseases. However, adding spatially structured extra-variability in the model fitted to the data when such extra-variability does not exist conditionally on the covariates included in the model (*over-fitting*) may bias the estimation of the ecological association between covariates and relative risks toward the null. In order to investigate that possibility, a simulation study of the impact of introducing unnecessary residual spatial structure in the estimation model was conducted.

**Results:**

In the case where no underlying extra-variability from the Poisson process exists, the simulation results show that models accounting for structured and unstructured residuals do not underestimate the ecological association, unless covariates have a very strong autocorrelation structure, i.e., 0.98 at 100 km on a territory of diameter 1000 km."

## 1 Background

Ecological regression studies investigate potential association between geographical variation in disease rates (or counts) and environmental covariates. For example, a recent study evaluated the ecological association between indoor radon concentration and acute leukaemia incidence among children [1]. For rare diseases and/or small areas, Bayesian hierarchical Poisson model is commonly used where within-area variability of disease is modelled at the first stage as a Poisson process and ecological relationships between disease and covariates are introduced at the second stage of the hierarchical model. Spatially extra-Poisson variability potentially due to aggregated effect of unknown confounders is commonly taken into account through spatially structured residuals added in the second stage of the model.

In that context, the BYM (Besag, York and Mollié) model [2] is a standard model for estimation of the ecological associations. The overall variability of a health indicator is broken down into a random Poisson component, a spatially structured area-specific random effect and an unstructured random term, across geographic units. It has been extensively shown that not accounting for an actual spatial variability may lead to major biases [3].

Conversely, if the spatial variability of a health indicator is completely explained by that of environmental factors and the other ecological covariates taken into consideration, regression residuals do not have spatial structure. Modelling the spatial structure of residuals could then lead to a biased estimate of the ecological association via a phenomenon of *over-fitting *[4-7].

To the author's knowledge, the quantitative impact of fitting a model including extra-Poisson variability to analyse data generated by a model where such extra-Poisson variability does not exist conditionally on the covariates included in the model (*over-fitting*) has not previously been explicitly and quantitatively investigated. Robustness of residuals modelling as BYM was studied in a different inferential context. In the frame of an extensive investigation of the statistical performances of a number of spatial models, Lawson *et al*. [8] studied the performance of such models on relative risks estimates in the case of mapping modelling, i.e. without covariates, where different true spatial structures of residuals were simulated. The authors showed that BYM model performed well on risks estimations except when the true residuals were resulting from a mixture structure. Different models were compared to detect effect from a putative source on a regular lattice [9]. They concluded in particular that the introduction of a spatially structured area-specific random effect leads to much less bias in the parameter estimate and that BYM model is the least biased. Notably, biases in parameter estimation appear when the random effects are not acounted for. In the present study we focused on ecological association estimate when covariates with spatially structure are introduced. Such covariates are often of interest in epidemiology when environmental exposures are studied. In our work we focus on a particular model with France mainland as study domain. The aim of our study was then to determine the robustness of the BYM model in the absence of residual spatial variation, i.e., the impact on the ecological association estimate on an irregular domain. The estimates performances were discussed and characterized according to the covariates structure. Simulations protocols assumed systematically a Poisson model at the first stage of the hierarchical model and log linear relationship between the incidence of the disease and exposure at the second stage without addition of extra-Poisson residuals. Various spatial structures of exposure were considered. The explained spatial variability is thus fully specified/attributable by the covariate structure.

First, ecological models that do or do not allow spatially structured or unstructured heterogeneity will be considered. Various simulation protocols for parameter values enabling balanced or unbalanced between/within area variability will then be presented. The results of the various simulation protocols will then be considered in terms of their performances with regard to the estimation of ecological associations. The paper will conclude with a discussion.

## 2 Methods

### 2.1 Statistical Models

Let *D *be the study area of interest, partitioned into *m *geographic areas. The data consist in the observed *Y*_*i *_and expected *E*_*i *_disease counts for each area *i*, (*i *= 1,...,*m*). Let *X*_*i *_be an ecological variable of interest in area *i*. The ecological Poisson *M*_0 _model is expressed in hierarchical form as follow:

Yi|Ri~Poisson (EiRi)log⁡(Ri)|α,β,Xi=α+βXi(M0)
 MathType@MTEF@5@5@+=feaafiart1ev1aaatCvAUfKttLearuWrP9MDH5MBPbIqV92AaeXatLxBI9gBaebbnrfifHhDYfgasaacH8akY=wiFfYdH8Gipec8Eeeu0xXdbba9frFj0=OqFfea0dXdd9vqai=hGuQ8kuc9pgc9s8qqaq=dirpe0xb9q8qiLsFr0=vr0=vr0dc8meaabaqaciaacaGaaeqabaqabeGadaaakeaafaqabeqacaaabaqbaeaabiWaaaqaaiabdMfaznaaBaaaleaacqWGPbqAaeqaaOGaeiiFaWNaemOuai1aaSbaaSqaaiabdMgaPbqabaaakeaacqGG+bGFaeaacqqGqbaucqqGVbWBcqqGPbqAcqqGZbWCcqqGZbWCcqqGVbWBcqqGUbGBcqqGGaaicqGGOaakcqWGfbqrdaWgaaWcbaGaemyAaKgabeaakiabdkfasnaaBaaaleaacqWGPbqAaeqaaOGaeiykaKcabaGagiiBaWMaei4Ba8Maei4zaCMaeiikaGIaemOuai1aaSbaaSqaaiabdMgaPbqabaGccqGGPaqkcqGG8baFiiGacqWFXoqycqGGSaalcqWFYoGycqGGSaalcqWGybawdaWgaaWcbaGaemyAaKgabeaaaOqaaiabg2da9aqaaiab=f7aHjabgUcaRiab=j7aIjabdIfaynaaBaaaleaacqWGPbqAaeqaaaaaaOqaaiabcIcaOiabd2eannaaBaaaleaacqaIWaamaeqaaOGaeiykaKcaaaaa@6476@

in which *R*_*i *_is the relative risk in area *i*. The second stage models the relationship between the relative risk and exposure variable. The ecological model *M*_0 _does not include any spatially structured or unstructured heterogeneity.

In order to account for those variabilities, the BYM model [2] was proposed :

Yi|Ri~Poisson(EiRi)log⁡(Ri)|α,β,Ui,Vi=α+βXi+Ui+ViUi|Uj≠i~N(∑j∈δiUj/ni,1/τU2ni)Vi|τV~N(0,τV2)(BYM)
 MathType@MTEF@5@5@+=feaafiart1ev1aaatCvAUfKttLearuWrP9MDH5MBPbIqV92AaeXatLxBI9gBaebbnrfifHhDYfgasaacH8akY=wiFfYdH8Gipec8Eeeu0xXdbba9frFj0=OqFfea0dXdd9vqai=hGuQ8kuc9pgc9s8qqaq=dirpe0xb9q8qiLsFr0=vr0=vr0dc8meaabaqaciaacaGaaeqabaqabeGadaaakeaafaqabeqacaaabaqbaeaabqWaaaaabaGaemywaK1aaSbaaSqaaiabdMgaPbqabaGccqGG8baFcqWGsbGudaWgaaWcbaGaemyAaKgabeaaaOqaaiabc6ha+bqaaiabbcfaqjabb+gaVjabbMgaPjabbohaZjabbohaZjabb+gaVjabb6gaUjabcIcaOiabdweafnaaBaaaleaacqWGPbqAaeqaaOGaemOuai1aaSbaaSqaaiabdMgaPbqabaGccqGGPaqkaeaacyGGSbaBcqGGVbWBcqGGNbWzcqGGOaakcqWGsbGudaWgaaWcbaGaemyAaKgabeaakiabcMcaPiabcYha8HGaciab=f7aHjabcYcaSiab=j7aIjabcYcaSiabdwfavnaaBaaaleaacqWGPbqAaeqaaOGaeiilaWIaemOvay1aaSbaaSqaaiabdMgaPbqabaaakeaacqGH9aqpaeaacqWFXoqycqGHRaWkcqWFYoGycqWGybawdaWgaaWcbaGaemyAaKgabeaakiabgUcaRiabdwfavnaaBaaaleaacqWGPbqAaeqaaOGaey4kaSIaemOvay1aaSbaaSqaaiabdMgaPbqabaaakeaacqWGvbqvdaWgaaWcbaGaemyAaKgabeaakiabcYha8jabdwfavnaaBaaaleaacqWGQbGAcqGHGjsUcqWGPbqAaeqaaaGcbaGaeiOFa4habaGaemOta4KaeiikaGYaaabeaeaacqWGvbqvdaWgaaWcbaGaemOAaOgabeaakiabc+caViabd6gaUnaaBaaaleaacqWGPbqAaeqaaOGaeiilaWIaeGymaeJaei4la8Iae8hXdq3aa0baaSqaaiabdwfavbqaaiabikdaYaaakiabd6gaUnaaBaaaleaacqWGPbqAaeqaaOGaeiykaKcaleaacqWGQbGAcqGHiiIZcqWF0oazdaWgaaadbaGaemyAaKgabeaaaSqab0GaeyyeIuoaaOqaaiabdAfawnaaBaaaleaacqWGPbqAaeqaaOGaeiiFaWNae8hXdq3aaSbaaSqaaiabdAfawbqabaaakeaacqGG+bGFaeaacqWGobGtcqGGOaakcqaIWaamcqGGSaalcqWFepaDdaqhaaWcbaGaemOvayfabaGaeGOmaidaaOGaeiykaKcaaaqaaiabcIcaOiabdkeacjabdMfazjabd2eanjabcMcaPaaaaaa@A873@

in which *δ*_*i *_denotes the set of labels of the neighbours of area *i*, *n*_*i *_is the number of neighbours *i*, *U*_*i *_(*i *= 1,...,*m*) models the spatially-structured area-specific random effect based on the conditional autoregressive approach CAR [10], and *V*_*i *_(*i *= 1,...,*m*) is the unstructured random effect. The BYM model is the benchmark parametric model and is widely used in disease-mapping studies mainly because of the flexibility of the residuals.

### 2.2 Design of the simulation study

Processes X and Y are simulated on D under model *M*_0 _accordingly to parameters values. The simulation parameters were selected with reference to the overall variability of the estimated relative risks, thus enabling realistic and reasonable values for relative risks. More precisely, let R^i
 MathType@MTEF@5@5@+=feaafiart1ev1aaatCvAUfKttLearuWrP9MDH5MBPbIqV92AaeXatLxBI9gBaebbnrfifHhDYfgasaacH8akY=wiFfYdH8Gipec8Eeeu0xXdbba9frFj0=OqFfea0dXdd9vqai=hGuQ8kuc9pgc9s8qqaq=dirpe0xb9q8qiLsFr0=vr0=vr0dc8meaabaqaciaacaGaaeqabaqabeGadaaakeaacuWGsbGugaqcamaaBaaaleaacqWGPbqAaeqaaaaa@2F70@ = *Y*_*i*_/*E*_*i *_be the maximum likelihood estimate of the relative risk for the area. If *X*_*i *_~ *N*(0, 1), then:

Var[log⁡(R^i)]≈E(1EiRi)+β2.
 MathType@MTEF@5@5@+=feaafiart1ev1aaatCvAUfKttLearuWrP9MDH5MBPbIqV92AaeXatLxBI9gBaebbnrfifHhDYfgasaacH8akY=wiFfYdH8Gipec8Eeeu0xXdbba9frFj0=OqFfea0dXdd9vqai=hGuQ8kuc9pgc9s8qqaq=dirpe0xb9q8qiLsFr0=vr0=vr0dc8meaabaqaciaacaGaaeqabaqabeGadaaakeaacqWGwbGvcqWGHbqycqWGYbGCdaWadaqaaiGbcYgaSjabc+gaVjabcEgaNjabcIcaOiqbdkfaszaajaWaaSbaaSqaaiabdMgaPbqabaGccqGGPaqkaiaawUfacaGLDbaacqGHijYUcqWGfbqrcqGGOaakdaWcaaqaaiabigdaXaqaaiabdweafnaaBaaaleaacqWGPbqAaeqaaOGaemOuai1aaSbaaSqaaiabdMgaPbqabaaaaOGaeiykaKIaey4kaSccciGae8NSdi2aaWbaaSqabeaacqaIYaGmaaGccqGGUaGlaaa@4A98@

If the relative risks are spatially independent of the expected disease counts *E*_*i*_,

E(1EiRi)≈1/E¯h×E(1/Ri),
 MathType@MTEF@5@5@+=feaafiart1ev1aaatCvAUfKttLearuWrP9MDH5MBPbIqV92AaeXatLxBI9gBaebbnrfifHhDYfgasaacH8akY=wiFfYdH8Gipec8Eeeu0xXdbba9frFj0=OqFfea0dXdd9vqai=hGuQ8kuc9pgc9s8qqaq=dirpe0xb9q8qiLsFr0=vr0=vr0dc8meaabaqaciaacaGaaeqabaqabeGadaaakeaacqWGfbqrcqGGOaakdaWcaaqaaiabigdaXaqaaiabdweafnaaBaaaleaacqWGPbqAaeqaaOGaemOuai1aaSbaaSqaaiabdMgaPbqabaaaaOGaeiykaKIaeyisISRaeGymaeJaei4la8IafmyrauKbaebadaWgaaWcbaGaemiAaGgabeaakiabgEna0kabdweafjabcIcaOiabigdaXiabc+caViabdkfasnaaBaaaleaacqWGPbqAaeqaaOGaeiykaKIaeiilaWcaaa@4664@

where E¯h
 MathType@MTEF@5@5@+=feaafiart1ev1aaatCvAUfKttLearuWrP9MDH5MBPbIqV92AaeXatLxBI9gBaebbnrfifHhDYfgasaacH8akY=wiFfYdH8Gipec8Eeeu0xXdbba9frFj0=OqFfea0dXdd9vqai=hGuQ8kuc9pgc9s8qqaq=dirpe0xb9q8qiLsFr0=vr0=vr0dc8meaabaqaciaacaGaaeqabaqabeGadaaakeaacuWGfbqrgaqeamaaBaaaleaacqWGObaAaeqaaaaa@2F5C@ is the harmonic mean of *E*_*i *_(*i *= 1,...,*m*). The overall variance may be expressed as:

Var[log⁡(R^i)]≈1/E¯h×exp⁡(−α+β2)+β2.
 MathType@MTEF@5@5@+=feaafiart1ev1aaatCvAUfKttLearuWrP9MDH5MBPbIqV92AaeXatLxBI9gBaebbnrfifHhDYfgasaacH8akY=wiFfYdH8Gipec8Eeeu0xXdbba9frFj0=OqFfea0dXdd9vqai=hGuQ8kuc9pgc9s8qqaq=dirpe0xb9q8qiLsFr0=vr0=vr0dc8meaabaqaciaacaGaaeqabaqabeGadaaakeaacqWGwbGvcqWGHbqycqWGYbGCdaWadaqaaiGbcYgaSjabc+gaVjabcEgaNjabcIcaOiqbdkfaszaajaWaaSbaaSqaaiabdMgaPbqabaGccqGGPaqkaiaawUfacaGLDbaacqGHijYUcqaIXaqmcqGGVaWlcuWGfbqrgaqeamaaBaaaleaacqWGObaAaeqaaOGaey41aqRagiyzauMaeiiEaGNaeiiCaaNaeiikaGIaeyOeI0ccciGae8xSdeMaey4kaSIae8NSdi2aaWbaaSqabeaacqaIYaGmaaGccqGGPaqkcqGHRaWkcqWFYoGydaahaaWcbeqaaiabikdaYaaakiabc6caUaaa@542C@

This variance may be broken down into a *Within area variability *term Wv = 1/E¯h
 MathType@MTEF@5@5@+=feaafiart1ev1aaatCvAUfKttLearuWrP9MDH5MBPbIqV92AaeXatLxBI9gBaebbnrfifHhDYfgasaacH8akY=wiFfYdH8Gipec8Eeeu0xXdbba9frFj0=OqFfea0dXdd9vqai=hGuQ8kuc9pgc9s8qqaq=dirpe0xb9q8qiLsFr0=vr0=vr0dc8meaabaqaciaacaGaaeqabaqabeGadaaakeaacuWGfbqrgaqeamaaBaaaleaacqWGObaAaeqaaaaa@2F5C@ × exp(-*α *+ *β*^2^) and a *Between area variability *term Bv = *β*^2^. Let *p *denote the proportion of between area variance, *p *= Bv/*Var*[log(R^i
 MathType@MTEF@5@5@+=feaafiart1ev1aaatCvAUfKttLearuWrP9MDH5MBPbIqV92AaeXatLxBI9gBaebbnrfifHhDYfgasaacH8akY=wiFfYdH8Gipec8Eeeu0xXdbba9frFj0=OqFfea0dXdd9vqai=hGuQ8kuc9pgc9s8qqaq=dirpe0xb9q8qiLsFr0=vr0=vr0dc8meaabaqaciaacaGaaeqabaqabeGadaaakeaacuWGsbGugaqcamaaBaaaleaacqWGPbqAaeqaaaaa@2F70@)], a high value of *p *corresponds to high between-area variability, that is a high amount of information with which to estimate the ecological link *β*. Hereafter, without any loss of generality, *α *will be considered equal to 0.

The geographic scale unit consisted in the 94 *Departements *of mainland France (Corsica excluded). The expected disease counts (*E*_*i*_) consist in the expected cases of acute leukaemia in children aged less than 15 years for the period 1990–1998 *in Departement i*. The cases were retrieved from the French National Registry of Childhood Leukaemia and Lymphoma [11]. The expected numbers ranged from 4.2 to 204, with a harmonic mean of 23.35. Scenarios in which the within-area variance was either doubled (E¯h
 MathType@MTEF@5@5@+=feaafiart1ev1aaatCvAUfKttLearuWrP9MDH5MBPbIqV92AaeXatLxBI9gBaebbnrfifHhDYfgasaacH8akY=wiFfYdH8Gipec8Eeeu0xXdbba9frFj0=OqFfea0dXdd9vqai=hGuQ8kuc9pgc9s8qqaq=dirpe0xb9q8qiLsFr0=vr0=vr0dc8meaabaqaciaacaGaaeqabaqabeGadaaakeaacuWGfbqrgaqeamaaBaaaleaacqWGObaAaeqaaaaa@2F5C@ = 46.6) or divided by 10 (E¯h
 MathType@MTEF@5@5@+=feaafiart1ev1aaatCvAUfKttLearuWrP9MDH5MBPbIqV92AaeXatLxBI9gBaebbnrfifHhDYfgasaacH8akY=wiFfYdH8Gipec8Eeeu0xXdbba9frFj0=OqFfea0dXdd9vqai=hGuQ8kuc9pgc9s8qqaq=dirpe0xb9q8qiLsFr0=vr0=vr0dc8meaabaqaciaacaGaaeqabaqabeGadaaakeaacuWGfbqrgaqeamaaBaaaleaacqWGObaAaeqaaaaa@2F5C@ = 2.33) were also considered.

Given that *X*_*i *_~ *N*(0, 1), within-area variance depends on 3 parameters, namely: the harmonic mean of expected disease counts E¯h
 MathType@MTEF@5@5@+=feaafiart1ev1aaatCvAUfKttLearuWrP9MDH5MBPbIqV92AaeXatLxBI9gBaebbnrfifHhDYfgasaacH8akY=wiFfYdH8Gipec8Eeeu0xXdbba9frFj0=OqFfea0dXdd9vqai=hGuQ8kuc9pgc9s8qqaq=dirpe0xb9q8qiLsFr0=vr0=vr0dc8meaabaqaciaacaGaaeqabaqabeGadaaakeaacuWGfbqrgaqeamaaBaaaleaacqWGObaAaeqaaaaa@2F5C@, the ecological link *β *and the autocorrelation structure of *X*_*i*_.

As *X *has a standardized normal distribution, the 2.5 (*p*_2.5%_) and 97.5 (*p*_97.5%_) percentiles of the relative risks are under model *M*_0 _exp(*α *± 1.96*β*) and their ratio *Q *= *p*_97.5%_/*p*_2.5% _is exp(2 × *β *× 1.96). The quantile ratios were considered equal to 1.0, 1.5, 2.0 and 3.0, equivalent to no effect, weak, moderate and strong effects, respectively. The corresponding values for *β *were 0.00, 0.12, 0.21 and 0.33. The proportions of between-area variance, *p*, by ecological link *β *and E¯h
 MathType@MTEF@5@5@+=feaafiart1ev1aaatCvAUfKttLearuWrP9MDH5MBPbIqV92AaeXatLxBI9gBaebbnrfifHhDYfgasaacH8akY=wiFfYdH8Gipec8Eeeu0xXdbba9frFj0=OqFfea0dXdd9vqai=hGuQ8kuc9pgc9s8qqaq=dirpe0xb9q8qiLsFr0=vr0=vr0dc8meaabaqaciaacaGaaeqabaqabeGadaaakeaacuWGfbqrgaqeamaaBaaaleaacqWGObaAaeqaaaaa@2F5C@ breakdown are summarized in Table 1.

**Table 1:** Between area variance proportion, *p*^(3)^, according to ecological link *β *and E¯h
 MathType@MTEF@5@5@+=feaafiart1ev1aaatCvAUfKttLearuWrP9MDH5MBPbIqV92AaeXatLxBI9gBaebbnrfifHhDYfgasaacH8akY=wiFfYdH8Gipec8Eeeu0xXdbba9frFj0=OqFfea0dXdd9vqai=hGuQ8kuc9pgc9s8qqaq=dirpe0xb9q8qiLsFr0=vr0=vr0dc8meaabaqaciaacaGaaeqabaqabeGadaaakeaacuWGfbqrgaqeamaaBaaaleaacqWGObaAaeqaaaaa@2F5C@

*β*^(1)^\E¯h(2) MathType@MTEF@5@5@+=feaafiart1ev1aaatCvAUfKttLearuWrP9MDH5MBPbIqV92AaeXatLxBI9gBaebbnrfifHhDYfgasaacH8akY=wiFfYdH8Gipec8Eeeu0xXdbba9frFj0=OqFfea0dXdd9vqai=hGuQ8kuc9pgc9s8qqaq=dirpe0xb9q8qiLsFr0=vr0=vr0dc8meaabaqaciaacaGaaeqabaqabeGadaaakeaacuWGfbqrgaqeamaaDaaaleaacqWGObaAaeaacqGGOaakcqaIYaGmcqGGPaqkaaaaaa@3201@	2.33	23.35	46.6
0.00	0.00	0.00	0.00
0.12	0.03	0.25	0.40
0.21	0.09	0.50	0.67
0.33	0.19	0.71	0.83

^(1)^*β*: Ecological link

^ (2)^E¯h
 MathType@MTEF@5@5@+=feaafiart1ev1aaatCvAUfKttLearuWrP9MDH5MBPbIqV92AaeXatLxBI9gBaebbnrfifHhDYfgasaacH8akY=wiFfYdH8Gipec8Eeeu0xXdbba9frFj0=OqFfea0dXdd9vqai=hGuQ8kuc9pgc9s8qqaq=dirpe0xb9q8qiLsFr0=vr0=vr0dc8meaabaqaciaacaGaaeqabaqabeGadaaakeaacuWGfbqrgaqeamaaBaaaleaacqWGObaAaeqaaaaa@2F5C@: Harmonic mean of expected disease counts

^(3)^*p *= Bv/*Var*[log(R^i
 MathType@MTEF@5@5@+=feaafiart1ev1aaatCvAUfKttLearuWrP9MDH5MBPbIqV92AaeXatLxBI9gBaebbnrfifHhDYfgasaacH8akY=wiFfYdH8Gipec8Eeeu0xXdbba9frFj0=OqFfea0dXdd9vqai=hGuQ8kuc9pgc9s8qqaq=dirpe0xb9q8qiLsFr0=vr0=vr0dc8meaabaqaciaacaGaaeqabaqabeGadaaakeaacuWGsbGugaqcamaaBaaaleaacqWGPbqAaeqaaaaa@2F70@)]: Between area variance proportion

^(*)^ : Indicator of p-value < 5% from McNemar's test (1)(2)(3) for comparing proportions 1 - *π*(0) under *M*_0 _and *BYM *models.

For E¯h
 MathType@MTEF@5@5@+=feaafiart1ev1aaatCvAUfKttLearuWrP9MDH5MBPbIqV92AaeXatLxBI9gBaebbnrfifHhDYfgasaacH8akY=wiFfYdH8Gipec8Eeeu0xXdbba9frFj0=OqFfea0dXdd9vqai=hGuQ8kuc9pgc9s8qqaq=dirpe0xb9q8qiLsFr0=vr0=vr0dc8meaabaqaciaacaGaaeqabaqabeGadaaakeaacuWGfbqrgaqeamaaBaaaleaacqWGObaAaeqaaaaa@2F5C@ = 23.35, the between area variance proportion ranges from 0 to 71%, with a balanced case for an ecological link when *β *= 0.21. For E¯h
 MathType@MTEF@5@5@+=feaafiart1ev1aaatCvAUfKttLearuWrP9MDH5MBPbIqV92AaeXatLxBI9gBaebbnrfifHhDYfgasaacH8akY=wiFfYdH8Gipec8Eeeu0xXdbba9frFj0=OqFfea0dXdd9vqai=hGuQ8kuc9pgc9s8qqaq=dirpe0xb9q8qiLsFr0=vr0=vr0dc8meaabaqaciaacaGaaeqabaqabeGadaaakeaacuWGfbqrgaqeamaaBaaaleaacqWGObaAaeqaaaaa@2F5C@ = 46.69, *p *ranges from 0 to 83%, while *p *ranges from 0 to 19% when E¯h
 MathType@MTEF@5@5@+=feaafiart1ev1aaatCvAUfKttLearuWrP9MDH5MBPbIqV92AaeXatLxBI9gBaebbnrfifHhDYfgasaacH8akY=wiFfYdH8Gipec8Eeeu0xXdbba9frFj0=OqFfea0dXdd9vqai=hGuQ8kuc9pgc9s8qqaq=dirpe0xb9q8qiLsFr0=vr0=vr0dc8meaabaqaciaacaGaaeqabaqabeGadaaakeaacuWGfbqrgaqeamaaBaaaleaacqWGObaAaeqaaaaa@2F5C@ = 2.33.

**Figure 1:**
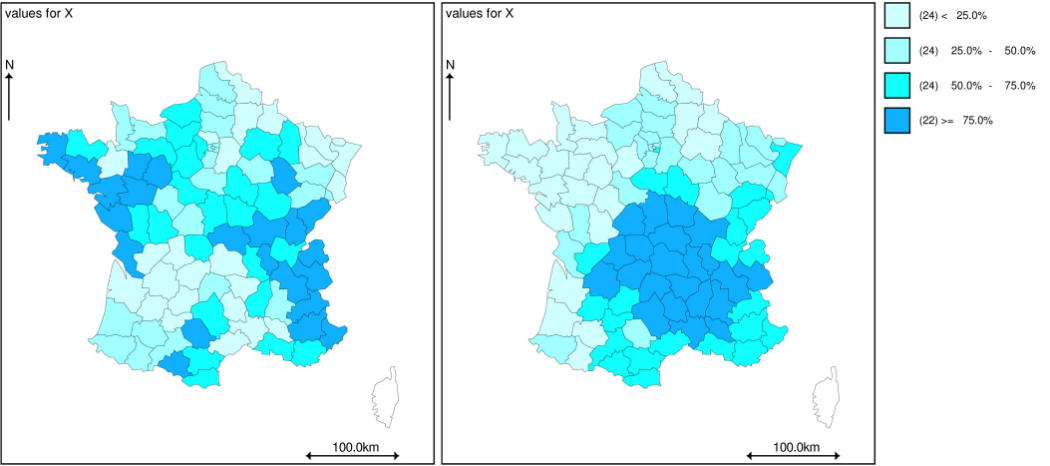
Replicates of a gaussian covariate with autocorrelation strenght of *ρ*_*xx *_= 0.80 (left) and *ρ*_*xx *_= 0.98 (right) at 100 km, (in quartiles).

The autocorrelation of the exposure variable was also modulated. The following exponential autocorrelation structure was considered: cov(*X*_*i*_, *X*_*j*_) = *exp*(-*d*(*i*, *j*)*φ*), in which *d*(*i, j*) is the distance between areas *i *and *j*. Let *ρ*_*xx *_= *exp*(-100*φ*) be the autocorrelation of two areas 100 km distant from each other. The following values for *ρ*_*xx *_= (0.40, 0.90,0.95, 0.98) were studied. That correlation structure is shown in Figures 1. High values of *ρ*_*xx *_may mimic a spatial bloc structure.

For each combination of parameters (E¯h
 MathType@MTEF@5@5@+=feaafiart1ev1aaatCvAUfKttLearuWrP9MDH5MBPbIqV92AaeXatLxBI9gBaebbnrfifHhDYfgasaacH8akY=wiFfYdH8Gipec8Eeeu0xXdbba9frFj0=OqFfea0dXdd9vqai=hGuQ8kuc9pgc9s8qqaq=dirpe0xb9q8qiLsFr0=vr0=vr0dc8meaabaqaciaacaGaaeqabaqabeGadaaakeaacuWGfbqrgaqeamaaBaaaleaacqWGObaAaeqaaaaa@2F5C@, *β*, *ρ*_*xx*_), 400 replicates of (*X*, *Y*) = ((*X*_*i*_, *Y*_*i*_), *i *= 1,...,*N*) were generated using the *M*_0 _model. For each replication, the ecological link was estimated by both models (*M*_0 _and BYM) in a Bayesian framework.

The estimations were made with BRugs [12] software. For each data set, a burn-in of 5000 iterations was used and Bayesian inferences were based on 45000 iterations from Gibbs sampling giving Monte Carlo standard errors of less than 5% of the posterior standard deviation of each parameter [13]. The Monte Carlo standard error is an estimate of the difference between the mean of the sampled values and the true posterior mean. Non-informative priors were chosen for the parameters: *α *~ *U*(-∞; +∞), *β *~ *N*(0.0, 1.0*E *+ 5), *τ*_*U *_~ Γ(0.5, 0.0005), *τ*_*V *_~ Γ(0.5, 0.0005) [14], in which Γ(*a*, *b*) denotes the Gamma distribution with expectation equal to *a/b*.

For each triplet (E¯h
 MathType@MTEF@5@5@+=feaafiart1ev1aaatCvAUfKttLearuWrP9MDH5MBPbIqV92AaeXatLxBI9gBaebbnrfifHhDYfgasaacH8akY=wiFfYdH8Gipec8Eeeu0xXdbba9frFj0=OqFfea0dXdd9vqai=hGuQ8kuc9pgc9s8qqaq=dirpe0xb9q8qiLsFr0=vr0=vr0dc8meaabaqaciaacaGaaeqabaqabeGadaaakeaacuWGfbqrgaqeamaaBaaaleaacqWGObaAaeqaaaaa@2F5C@, *β*, *ρ*_*xx*_), the ecological link was estimated using the *M*_0 _and BYM models. Let β^(j)
 MathType@MTEF@5@5@+=feaafiart1ev1aaatCvAUfKttLearuWrP9MDH5MBPbIqV92AaeXatLxBI9gBaebbnrfifHhDYfgasaacH8akY=wiFfYdH8Gipec8Eeeu0xXdbba9frFj0=OqFfea0dXdd9vqai=hGuQ8kuc9pgc9s8qqaq=dirpe0xb9q8qiLsFr0=vr0=vr0dc8meaabaqaciaacaGaaeqabaqabeGadaaakeaaiiGacuWFYoGygaqcamaaCaaaleqabaGaeiikaGIaemOAaOMaeiykaKcaaaaa@31A0@ be the posterior mean estimates of *β *at the *j*th replication, σβ^(j)
 MathType@MTEF@5@5@+=feaafiart1ev1aaatCvAUfKttLearuWrP9MDH5MBPbIqV92AaeXatLxBI9gBaebbnrfifHhDYfgasaacH8akY=wiFfYdH8Gipec8Eeeu0xXdbba9frFj0=OqFfea0dXdd9vqai=hGuQ8kuc9pgc9s8qqaq=dirpe0xb9q8qiLsFr0=vr0=vr0dc8meaabaqaciaacaGaaeqabaqabeGadaaakeaaiiGacqWFdpWCdaWgaaWcbaGaf8NSdiMbaKaadaahaaadbeqaaiabcIcaOiabdQgaQjabcMcaPaaaaSqabaaaaa@3396@ the posterior standard error of β^(j)
 MathType@MTEF@5@5@+=feaafiart1ev1aaatCvAUfKttLearuWrP9MDH5MBPbIqV92AaeXatLxBI9gBaebbnrfifHhDYfgasaacH8akY=wiFfYdH8Gipec8Eeeu0xXdbba9frFj0=OqFfea0dXdd9vqai=hGuQ8kuc9pgc9s8qqaq=dirpe0xb9q8qiLsFr0=vr0=vr0dc8meaabaqaciaacaGaaeqabaqabeGadaaakeaaiiGacuWFYoGygaqcamaaCaaaleqabaGaeiikaGIaemOAaOMaeiykaKcaaaaa@31A0@ and *CI*^*j*^(*β*) the 95% credibility interval of *β*. The following criteria were computed for 400 replications (*j *= 1,...,400):

• The empirical mean of the estimated posterior expectations of β(β¯sim=∑j=1400β^(j)/400)
 MathType@MTEF@5@5@+=feaafiart1ev1aaatCvAUfKttLearuWrP9MDH5MBPbIqV92AaeXatLxBI9gBaebbnrfifHhDYfgasaacH8akY=wiFfYdH8Gipec8Eeeu0xXdbba9frFj0=OqFfea0dXdd9vqai=hGuQ8kuc9pgc9s8qqaq=dirpe0xb9q8qiLsFr0=vr0=vr0dc8meaabaqaciaacaGaaeqabaqabeGadaaakeaaiiGacqWFYoGydaqadaqaaiqb=j7aIzaaraWaaSbaaSqaaiabdohaZjabdMgaPjabd2gaTbqabaGccqGH9aqpdaaeWaqaaiqb=j7aIzaajaWaaWbaaSqabeaacqGGOaakcqWGQbGAcqGGPaqkaaGccqGGVaWlcqaI0aancqaIWaamcqaIWaamaSqaaiabdQgaQjabg2da9iabigdaXaqaaiabisda0iabicdaWiabicdaWaqdcqGHris5aaGccaGLOaGaayzkaaaaaa@47D5@

• The empirical mean of the estimated posterior standard deviations of β(σ¯βsim=∑j=1400σβ^(j)/400)
 MathType@MTEF@5@5@+=feaafiart1ev1aaatCvAUfKttLearuWrP9MDH5MBPbIqV92AaeXatLxBI9gBaebbnrfifHhDYfgasaacH8akY=wiFfYdH8Gipec8Eeeu0xXdbba9frFj0=OqFfea0dXdd9vqai=hGuQ8kuc9pgc9s8qqaq=dirpe0xb9q8qiLsFr0=vr0=vr0dc8meaabaqaciaacaGaaeqabaqabeGadaaakeaaiiGacqWFYoGydaqadaqaaiqb=n8aZzaaraWaaSbaaSqaaiab=j7aInaaBaaameaacqWGZbWCcqWGPbqAcqWGTbqBaeqaaaWcbeaakiabg2da9maaqadabaGae83Wdm3aaSbaaSqaaiqb=j7aIzaajaWaaWbaaWqabeaacqGGOaakcqWGQbGAcqGGPaqkaaaaleqaaOGaei4la8IaeGinaqJaeGimaaJaeGimaadaleaacqWGQbGAcqGH9aqpcqaIXaqmaeaacqaI0aancqaIWaamcqaIWaama0GaeyyeIuoaaOGaayjkaiaawMcaaaaa@4BC1@

• The empirical standard deviation of the 400 estimated posterior means of *β *(*sd*(*β*_*sim*_))

• The mean relative bias (MB) and its standard deviation (sd(MB)),

• The root mean square error (RMSE),

• The proportion of coverage, *π*(*β*): the percentage of time when *β *lay within its 95% credibility interval

• The proportion of non-coverage 1 - *π*(0): the percentage of time when 0 did not lie within its 95% credibility interval

When *β *≠ 0, 1 - *π*(0) quantifies the ability of the estimation model to detect the existence of an association, which is analogous to the frequentist power. McNemar's test for comparing proportions from paired data (estimates of *β *from *M*_0 _and BYM based on the same replicated data set) was used to test whether the *π*(*β*) (or 1 - *π*(0)) values were significantly different. The over-fit of the BYM model (compared to the *M*_0 _model) was assessed via that criterion.

## 3 Simulation Results

Simulations results are structured as follow: firstly, we present results for an harmonic mean E¯h
 MathType@MTEF@5@5@+=feaafiart1ev1aaatCvAUfKttLearuWrP9MDH5MBPbIqV92AaeXatLxBI9gBaebbnrfifHhDYfgasaacH8akY=wiFfYdH8Gipec8Eeeu0xXdbba9frFj0=OqFfea0dXdd9vqai=hGuQ8kuc9pgc9s8qqaq=dirpe0xb9q8qiLsFr0=vr0=vr0dc8meaabaqaciaacaGaaeqabaqabeGadaaakeaacuWGfbqrgaqeamaaBaaaleaacqWGObaAaeqaaaaa@2F5C@ = 23.35 of expected disease counts equal to those from acute leukaemia in children for 1990–1998 in France and a covariate *X *with null to moderate autocorrelation. Secondly, for the same harmonic mean E¯h
 MathType@MTEF@5@5@+=feaafiart1ev1aaatCvAUfKttLearuWrP9MDH5MBPbIqV92AaeXatLxBI9gBaebbnrfifHhDYfgasaacH8akY=wiFfYdH8Gipec8Eeeu0xXdbba9frFj0=OqFfea0dXdd9vqai=hGuQ8kuc9pgc9s8qqaq=dirpe0xb9q8qiLsFr0=vr0=vr0dc8meaabaqaciaacaGaaeqabaqabeGadaaakeaacuWGfbqrgaqeamaaBaaaleaacqWGObaAaeqaaaaa@2F5C@, we study the influence of strong autocorrelations for the covariate X and finally, variations of E¯h
 MathType@MTEF@5@5@+=feaafiart1ev1aaatCvAUfKttLearuWrP9MDH5MBPbIqV92AaeXatLxBI9gBaebbnrfifHhDYfgasaacH8akY=wiFfYdH8Gipec8Eeeu0xXdbba9frFj0=OqFfea0dXdd9vqai=hGuQ8kuc9pgc9s8qqaq=dirpe0xb9q8qiLsFr0=vr0=vr0dc8meaabaqaciaacaGaaeqabaqabeGadaaakeaacuWGfbqrgaqeamaaBaaaleaacqWGObaAaeqaaaaa@2F5C@ (smaller and greater than 23.35) are explored.

### 3.1 Moderate covariate autocorrelation

The first scenario considered E¯h
 MathType@MTEF@5@5@+=feaafiart1ev1aaatCvAUfKttLearuWrP9MDH5MBPbIqV92AaeXatLxBI9gBaebbnrfifHhDYfgasaacH8akY=wiFfYdH8Gipec8Eeeu0xXdbba9frFj0=OqFfea0dXdd9vqai=hGuQ8kuc9pgc9s8qqaq=dirpe0xb9q8qiLsFr0=vr0=vr0dc8meaabaqaciaacaGaaeqabaqabeGadaaakeaacuWGfbqrgaqeamaaBaaaleaacqWGObaAaeqaaaaa@2F5C@ = 23.35 and the autocorrelation equal to 0.0 or 0.4; the results are shown in Table 2. In that setting, the between-area variance varied from 0 to 71%. In the absence of any spatial structure for *X *(*ρ*_*xx *_= 0), the estimate of *β *was unbiased, irrespective of the estimation model. The mean bias was less than 1% and the RMSE was less than 0.02 for the four values of the ecological link (for both models). The coverage proportions *π*(*β*) were similar for the two models and greater than 94.5%. The coverage proportion of the BYM model was consistently slightly greater than that of the *M*_0 _model. For *β *= 0, *π*(*β*) was close to 95% and, equivalently, 1 - *π*(0) was close to 5%. The BYM model thus handles the scenario in which the covariate has no spatial structure.

**Table 2:** Estimation of the ecological link *β *when E¯h
 MathType@MTEF@5@5@+=feaafiart1ev1aaatCvAUfKttLearuWrP9MDH5MBPbIqV92AaeXatLxBI9gBaebbnrfifHhDYfgasaacH8akY=wiFfYdH8Gipec8Eeeu0xXdbba9frFj0=OqFfea0dXdd9vqai=hGuQ8kuc9pgc9s8qqaq=dirpe0xb9q8qiLsFr0=vr0=vr0dc8meaabaqaciaacaGaaeqabaqabeGadaaakeaacuWGfbqrgaqeamaaBaaaleaacqWGObaAaeqaaaaa@2F5C@ = 23.35 and *ρ*_*xx *_= 0.0, 0.4 (400 replications)

*ρ*_*xx*_^(1)^	*β*	Model	β¯ MathType@MTEF@5@5@+=feaafiart1ev1aaatCvAUfKttLearuWrP9MDH5MBPbIqV92AaeXatLxBI9gBaebbnrfifHhDYfgasaacH8akY=wiFfYdH8Gipec8Eeeu0xXdbba9frFj0=OqFfea0dXdd9vqai=hGuQ8kuc9pgc9s8qqaq=dirpe0xb9q8qiLsFr0=vr0=vr0dc8meaabaqaciaacaGaaeqabaqabeGadaaakeaaiiGacuWFYoGygaqeaaaa@2E6C@_*sim*_^(2)^	σ¯βsim MathType@MTEF@5@5@+=feaafiart1ev1aaatCvAUfKttLearuWrP9MDH5MBPbIqV92AaeXatLxBI9gBaebbnrfifHhDYfgasaacH8akY=wiFfYdH8Gipec8Eeeu0xXdbba9frFj0=OqFfea0dXdd9vqai=hGuQ8kuc9pgc9s8qqaq=dirpe0xb9q8qiLsFr0=vr0=vr0dc8meaabaqaciaacaGaaeqabaqabeGadaaakeaaiiGacuWFdpWCgaqeamaaBaaaleaacqWFYoGydaWgaaadbaGaem4CamNaemyAaKMaemyBa0gabeaaaSqabaaaaa@34BB@	MB^(4)^	sd(MB)	sd(*β*_*sim*_)^(5)^	RMSE^(6)^	*π*(*β*)^(7)^	1 - *π*(0)^(8)^
0.0	0.00	*M*_0_	0.000	1.63			1.73	1.73	0.953	0.047
		BYM	0.000	1.76			1.74	1.73	0.958	0.042
	0.12	*M*_0_	0.119	1.61	-0.44	0.69	1.67	1.67	0.930	1.000
		BYM	0.120	1.72	-0.31	0.70	1.68	1.68	0.955	1.000
	0.21	*M*_0_	0.210	1.60	0.49	0.39	1.64	1.64	0.948	1.000
		BYM	0.210	1.72	0.58	0.39	1.66	1.66	0.960	1.000
	0.33	*M*_0_	0.329	1.57	-0.28	0.23	1.58	1.58	0.953	1.000
		BYM	0.329	1.70	-0.24	0.24	1.58	1.58	0.968	1.000

0.4	0.00	*M*_0_	-0.001	1.72			1.76	1.76	0.955	0.045*
		BYM	-0.001	1.90			1.78	1.78	0.975	0.025
	0.12	*M*_0_	0.121	1.73	0.79	0.73	1.75	1.75	0.945	1.000
		BYM	0.121	1.91	0.78	0.74	1.78	1.78	0.968	1.000
	0.21	*M*_0_	0.211	1.72	0.41	0.39	1.66	1.66	0.958	1.000
		BYM	0.211	1.90	0.50	0.39	1.67	1.67	0.973	1.000
	0.33	*M*_0_	0.332	1.70	0.71	0.25	1.68	1.69	0.945	1.000
		BYM	0.332	1.89	0.70	0.25	1.70	1.71	0.973	1.000

^(1) ^*ρ*_*xx *_: autocorrelation at 100 km

^(2) ^β¯
 MathType@MTEF@5@5@+=feaafiart1ev1aaatCvAUfKttLearuWrP9MDH5MBPbIqV92AaeXatLxBI9gBaebbnrfifHhDYfgasaacH8akY=wiFfYdH8Gipec8Eeeu0xXdbba9frFj0=OqFfea0dXdd9vqai=hGuQ8kuc9pgc9s8qqaq=dirpe0xb9q8qiLsFr0=vr0=vr0dc8meaabaqaciaacaGaaeqabaqabeGadaaakeaaiiGacuWFYoGygaqeaaaa@2E6C@_*sim *_: 100 * mean of posterior means

^(3) ^σ¯βsim
 MathType@MTEF@5@5@+=feaafiart1ev1aaatCvAUfKttLearuWrP9MDH5MBPbIqV92AaeXatLxBI9gBaebbnrfifHhDYfgasaacH8akY=wiFfYdH8Gipec8Eeeu0xXdbba9frFj0=OqFfea0dXdd9vqai=hGuQ8kuc9pgc9s8qqaq=dirpe0xb9q8qiLsFr0=vr0=vr0dc8meaabaqaciaacaGaaeqabaqabeGadaaakeaaiiGacuWFdpWCgaqeamaaBaaaleaacqWFYoGydaWgaaadbaGaem4CamNaemyAaKMaemyBa0gabeaaaSqabaaaaa@34BB@ : 100 * mean of posterior standard deviations

^(4)^ MB : 100 * Mean Bias

^(5)^ sd(*β*_*sim*_) : 100 * standard deviation of posterior means

^(6)^ RMSE : 100 * Root Mean Square Error

^(7) ^*π*(*β*) : Coverage proportion *β ∈ CI*(*β*)

^(8)^ 1 - *π*(0) : Non-Coverage proportion 0 ∉ *CI*(*β*)

^(*)^ : Indicator of p-value < 5% from McNemar's test (1)(2)(3) for comparing proportions 1 - *π*(0) under *M*_0 _and *BYM *models.

When the autocorrelation was increased to 0.4, the results were similar to those with the previous setting. The mean bias was less than 1%. There was a slight increase in the RMSE but it remained less than 0.02. The *β *coverage proportion with the BYM model was greater than that with the *M*_0 _model. The non-coverage proportion was equal to 1, except when there was no association (*β *= 0). The non-coverage proportions were significantly different when *β *= 0. The proportion the closest to 5% was obtained with the *M*_0 _model. Irrespective of the value of *β*, the variability of *β *was always slightly over-estimated with the BYM model. The coverage proportion of the *M*_0 _model varied from 94.5 to 95.8% (96.8 to 97.5% for the BYM model). In the absence of, or with moderate, autocorrelation, the *over-fitting *effect was not observed. Both models provided an almost unbiased estimate of the ecological link.

### 3.2 Strong covariate autocorrelation

The scenario of strong autocorrelation for *X *was then considered: *ρ*_*xx *_= 0.90, 0.95, 0.98 at 100 km with E¯h
 MathType@MTEF@5@5@+=feaafiart1ev1aaatCvAUfKttLearuWrP9MDH5MBPbIqV92AaeXatLxBI9gBaebbnrfifHhDYfgasaacH8akY=wiFfYdH8Gipec8Eeeu0xXdbba9frFj0=OqFfea0dXdd9vqai=hGuQ8kuc9pgc9s8qqaq=dirpe0xb9q8qiLsFr0=vr0=vr0dc8meaabaqaciaacaGaaeqabaqabeGadaaakeaacuWGfbqrgaqeamaaBaaaleaacqWGObaAaeqaaaaa@2F5C@ = 23.35. The results are shown in Table 3. When *ρ*_*xx *_= 0.90, the mean bias and the RMSE were low (0.03). The mean bias decreased as the value of the ecological link increased, reflecting an increase in between-area variability. The coverage proportion with the BYM model was higher than the coverage proportion with the *M*_0 _model for all values of *β*. The non-coverage proportions were significantly different for *β *= 0.00 and *β *= 0.12, in favor of the *M*_0 _model. For *ρ*_*xx *_= 0.95, the bias was still small and the RMSE increased to 0.04. The non-coverage proportions were again significantly different in the cases in which *β *= 0.00 and *β *= 0.12 in favor of the *M*_0 _model. Lastly, for *ρ*_*xx *_= 0.98 and for the first three values of *β *(*β *= 0.00, 0.12, 0.21), the non-coverage proportions were significantly different, again in favor of *M*_0_. When the ecological link was null, the non-coverage proportion was again smaller with the BYM model, a consequence of the over-estimation of parameter variability. The *β *coverage proportions were lower for *M*_0 _for 4 values of *β*. When the autocorrelation increased from 0.90 to 0.98, the RMSE increased from 2.99 to 6.48, mainly due to the decrease in independent information. A slight increase was observed for all the other criteria. The bias was weak, resulting in very small RMSE and *sd*(*β*_*sim*_) in both models. At high autocorrelation values, the overall variability of the estimates increased. There was more variability for each *β *value with the BYM model. This is exemplified by the mean posterior standard deviation, which increased four-fold (for all *β *values) between the first (*ρ*_*xx *_= 0.00) and last (*ρ*_*xx *_= 0.98) autocorrelation scenario. This was also the case for *sd*(*β*_*sim*_).

**Table 3:** Estimation of the ecological link *β *when E¯h
 MathType@MTEF@5@5@+=feaafiart1ev1aaatCvAUfKttLearuWrP9MDH5MBPbIqV92AaeXatLxBI9gBaebbnrfifHhDYfgasaacH8akY=wiFfYdH8Gipec8Eeeu0xXdbba9frFj0=OqFfea0dXdd9vqai=hGuQ8kuc9pgc9s8qqaq=dirpe0xb9q8qiLsFr0=vr0=vr0dc8meaabaqaciaacaGaaeqabaqabeGadaaakeaacuWGfbqrgaqeamaaBaaaleaacqWGObaAaeqaaaaa@2F5C@ = 23.35 and *ρ*_*xx *_= 0.90, 0.95, 0.98

(1)(2)(3)(4)(5)(6)(7)(8)(*)										
*ρ*_*xx*_^(1)^	*β*	Model	β¯ MathType@MTEF@5@5@+=feaafiart1ev1aaatCvAUfKttLearuWrP9MDH5MBPbIqV92AaeXatLxBI9gBaebbnrfifHhDYfgasaacH8akY=wiFfYdH8Gipec8Eeeu0xXdbba9frFj0=OqFfea0dXdd9vqai=hGuQ8kuc9pgc9s8qqaq=dirpe0xb9q8qiLsFr0=vr0=vr0dc8meaabaqaciaacaGaaeqabaqabeGadaaakeaaiiGacuWFYoGygaqeaaaa@2E6C@_*sim*_^(2)^	σ¯βsim MathType@MTEF@5@5@+=feaafiart1ev1aaatCvAUfKttLearuWrP9MDH5MBPbIqV92AaeXatLxBI9gBaebbnrfifHhDYfgasaacH8akY=wiFfYdH8Gipec8Eeeu0xXdbba9frFj0=OqFfea0dXdd9vqai=hGuQ8kuc9pgc9s8qqaq=dirpe0xb9q8qiLsFr0=vr0=vr0dc8meaabaqaciaacaGaaeqabaqabeGadaaakeaaiiGacuWFdpWCgaqeamaaBaaaleaacqWFYoGydaWgaaadbaGaem4CamNaemyAaKMaemyBa0gabeaaaSqabaaaaa@34BB@	MB^(4)^	sd(MB)	sd(*β*_*sim*_)^(5)^	RMSE^(6)^	*π*(*β*)^(7)^	1 - *π*(0)^(8)^
0.90	0.00	*M*_0_	0.003	2.96			2.98	2.99	0.945	0.055*
		BYM	0.003	3.39			3.03	3.04	0.970	0.030
	0.12	*M*_0_	0.122	2.98	2.07	1.24	2.99	3.00	0.958	0.973*
		BYM	0.123	3.44	2.51	1.27	3.07	3.08	0.973	0.938
	0.21	*M*_0_	0.213	2.96	1.38	0.79	3.32	3.34	0.930	0.998
		BYM	0.213	3.41	1.31	0.81	3.41	3.42	0.955	0.998
	0.33	*M*_0_	0.331	2.98	0.37	0.47	3.11	3.11	0.943	1.000
		BYM	0.331	3.43	0.39	0.47	3.13	3.13	0.965	1.000

0.95	0.00	*M*_0_	0.002	4.07			4.04	4.04	0.950	0.050*
		BYM	0.002	4.72			4.14	4.14	0.975	0.025
	0.12	*M*_0_	0.121	4.05	1.03	1.82	4.38	4.37	0.945	0.843*
		BYM	0.121	4.70	1.18	1.89	4.54	4.54	0.960	0.775
	0.21	*M*_0_	0.208	3.99	-1.07	0.99	4.18	4.18	0.960	0.988
		BYM	0.208	4.62	-0.80	1.03	4.31	4.31	0.978	0.983
	0.33	*M*_0_	0.333	4.16	0.78	0.66	4.33	4.33	0.963	1.000
		BYM	0.333	4.80	0.98	0.66	4.34	4.35	0.980	1.000

0.98	0.00	*M*_0_	0.002	6.29			6.48	6.48	0.950	0.050*
		BYM	0.002	7.29			6.53	6.53	0.970	0.030
	0.12	*M*_0_	0.121	6.23	1.22	2.49	5.98	5.98	0.953	0.545*
		BYM	0.122	7.20	1.26	2.47	5.99	5.98	0.975	0.420
	0.21	*M*_0_	0.207	6.35	-1.36	1.57	6.61	6.60	0.960	0.845*
		BYM	0.207	7.35	-1.39	1.59	6.67	6.67	0.985	0.782
	0.33	*M*_0_	0.330	6.27	0.09	1.00	6.64	6.63	0.935	0.985
		BYM	0.331	7.28	0.24	1.01	6.69	6.68	0.975	0.978

^(1) ^*ρ*_*xx *_: autocorrelation at 100 km

^(2) ^β¯
 MathType@MTEF@5@5@+=feaafiart1ev1aaatCvAUfKttLearuWrP9MDH5MBPbIqV92AaeXatLxBI9gBaebbnrfifHhDYfgasaacH8akY=wiFfYdH8Gipec8Eeeu0xXdbba9frFj0=OqFfea0dXdd9vqai=hGuQ8kuc9pgc9s8qqaq=dirpe0xb9q8qiLsFr0=vr0=vr0dc8meaabaqaciaacaGaaeqabaqabeGadaaakeaaiiGacuWFYoGygaqeaaaa@2E6C@_*sim *_: 100 * mean of posterior means

^(3) ^σ¯βsim
 MathType@MTEF@5@5@+=feaafiart1ev1aaatCvAUfKttLearuWrP9MDH5MBPbIqV92AaeXatLxBI9gBaebbnrfifHhDYfgasaacH8akY=wiFfYdH8Gipec8Eeeu0xXdbba9frFj0=OqFfea0dXdd9vqai=hGuQ8kuc9pgc9s8qqaq=dirpe0xb9q8qiLsFr0=vr0=vr0dc8meaabaqaciaacaGaaeqabaqabeGadaaakeaaiiGacuWFdpWCgaqeamaaBaaaleaacqWFYoGydaWgaaadbaGaem4CamNaemyAaKMaemyBa0gabeaaaSqabaaaaa@34BB@ : 100 * mean of posterior standard deviations

^(4)^ MB : 100 * Mean Bias

^(5)^ sd(*β*_*sim*_) : 100 * standard deviation of posterior means

^(6)^ RMSE : 100 * Root Mean Square Error

^(7) ^*π*(*β*) : Coverage proportion *β ∈ CI*(*β*)

^(8)^ 1 - *π*(0) : Non-Coverage proportion 0 ∉ *CI*(*β*)

^(*)^ : Indicator of p-value < 5% from McNemar's test (1)(2)(3) for comparing proportions 1 - *π*(0) under *M*_0 _and *BYM *models.

### 3.3 Variation of (harmonic mean of) expected counts

The next scenario consisted in strong autocorrelation of *ρ*_*xx *_= 0.95 at 100 km with variation in number of expected disease counts. The results for that scenario are shown in Table 4.

**Table 4:** Estimation of the ecological link *β *when ^*ρ*^_*xx *_= 0.95 while E¯h
 MathType@MTEF@5@5@+=feaafiart1ev1aaatCvAUfKttLearuWrP9MDH5MBPbIqV92AaeXatLxBI9gBaebbnrfifHhDYfgasaacH8akY=wiFfYdH8Gipec8Eeeu0xXdbba9frFj0=OqFfea0dXdd9vqai=hGuQ8kuc9pgc9s8qqaq=dirpe0xb9q8qiLsFr0=vr0=vr0dc8meaabaqaciaacaGaaeqabaqabeGadaaakeaacuWGfbqrgaqeamaaBaaaleaacqWGObaAaeqaaaaa@2F5C@ varying

(1)(2)(3)(4)(5)(6)(7)(8)(*)										
E¯h MathType@MTEF@5@5@+=feaafiart1ev1aaatCvAUfKttLearuWrP9MDH5MBPbIqV92AaeXatLxBI9gBaebbnrfifHhDYfgasaacH8akY=wiFfYdH8Gipec8Eeeu0xXdbba9frFj0=OqFfea0dXdd9vqai=hGuQ8kuc9pgc9s8qqaq=dirpe0xb9q8qiLsFr0=vr0=vr0dc8meaabaqaciaacaGaaeqabaqabeGadaaakeaacuWGfbqrgaqeamaaBaaaleaacqWGObaAaeqaaaaa@2F5C@	*β*	Model	β¯ MathType@MTEF@5@5@+=feaafiart1ev1aaatCvAUfKttLearuWrP9MDH5MBPbIqV92AaeXatLxBI9gBaebbnrfifHhDYfgasaacH8akY=wiFfYdH8Gipec8Eeeu0xXdbba9frFj0=OqFfea0dXdd9vqai=hGuQ8kuc9pgc9s8qqaq=dirpe0xb9q8qiLsFr0=vr0=vr0dc8meaabaqaciaacaGaaeqabaqabeGadaaakeaaiiGacuWFYoGygaqeaaaa@2E6C@_*sim*_^(1)^	σ¯βsim MathType@MTEF@5@5@+=feaafiart1ev1aaatCvAUfKttLearuWrP9MDH5MBPbIqV92AaeXatLxBI9gBaebbnrfifHhDYfgasaacH8akY=wiFfYdH8Gipec8Eeeu0xXdbba9frFj0=OqFfea0dXdd9vqai=hGuQ8kuc9pgc9s8qqaq=dirpe0xb9q8qiLsFr0=vr0=vr0dc8meaabaqaciaacaGaaeqabaqabeGadaaakeaaiiGacuWFdpWCgaqeamaaBaaaleaacqWFYoGydaWgaaadbaGaem4CamNaemyAaKMaemyBa0gabeaaaSqabaaaaa@34BB@	MB^(3)^	sd(MB)	sd(*β*_*sim*_)^(4)^	RMSE^(5)^	*π*(*β*)^(6)^	1 - *π*(0)^(7)^
46.6	0.00	*M*_0_	-0.001	2.84			2.76	2.76	0.958	0.042*
		BYM	-0.001	3.40			2.85	2.85	0.975	0.025
	0.12	*M*_0_	0.118	2.81	-1.34	1.21	2.91	2.91	0.945	0.960*
		BYM	0.119	3.38	-1.18	1.21	2.91	2.91	0.980	0.930
	0.21	*M*_0_	0.212	2.87	0.94	0.69	2.89	2.89	0.955	1.000
		BYM	0.212	3.47	0.75	0.70	2.95	2.96	0.983	1.000
	0.33	*M*_0_	0.329	2.88	-0.21	0.47	3.10	3.10	0.953	1.000
		BYM	0.330	3.45	-0.08	0.48	3.19	3.19	0.980	1.000

23.3	0.00	*M*_0_	0.002	4.07			4.04	4.04	0.950	0.050*
		BYM	0.002	4.72			4.14	4.14	0.975	0.025
	0.12	*M*_0_	0.121	4.05	1.03	1.82	4.38	4.37	0.945	0.843*
		BYM	0.121	4.70	1.18	1.89	4.54	4.54	0.960	0.775
	0.21	*M*_0_	0.208	3.99	-1.07	0.99	4.18	4.18	0.960	0.988
		BYM	0.208	4.62	-0.80	1.03	4.31	4.31	0.978	0.983
	0.33	*M*_0_	0.333	4.16	0.78	0.66	4.33	4.34	0.963	1.000
		BYM	0.333	4.79	0.98	0.66	4.34	4.35	0.980	1.000

2.33	0.00	*M*_0_	0.007	12.4			13.5	13.4	0.935	0.065
		BYM	0.008	13.3			13.4	13.4	0.948	0.052
	0.12	*M*_0_	0.112	12.5	-6.94	5.38	12.9	12.9	0.953	0.162*
		BYM	0.113	13.4	-5.65	5.43	13.0	13.0	0.965	0.130
	0.21	*M*_0_	0.206	12.8	-1.92	3.45	14.5	14.5	0.927	0.410*
		BYM	0.206	13.8	-1.98	3.46	14.6	14.5	0.940	0.368
	0.33	*M*_0_	0.341	13.0	3.38	2.12	14.00	14.0	0.938	0.738*
		BYM	0.343	13.9	4.05	2.14	14.1	14.2	0.960	0.715

^(1)^β¯
 MathType@MTEF@5@5@+=feaafiart1ev1aaatCvAUfKttLearuWrP9MDH5MBPbIqV92AaeXatLxBI9gBaebbnrfifHhDYfgasaacH8akY=wiFfYdH8Gipec8Eeeu0xXdbba9frFj0=OqFfea0dXdd9vqai=hGuQ8kuc9pgc9s8qqaq=dirpe0xb9q8qiLsFr0=vr0=vr0dc8meaabaqaciaacaGaaeqabaqabeGadaaakeaaiiGacuWFYoGygaqeaaaa@2E6C@_*sim *_: 100 * mean of posterior means

^(2)^σ¯βsim
 MathType@MTEF@5@5@+=feaafiart1ev1aaatCvAUfKttLearuWrP9MDH5MBPbIqV92AaeXatLxBI9gBaebbnrfifHhDYfgasaacH8akY=wiFfYdH8Gipec8Eeeu0xXdbba9frFj0=OqFfea0dXdd9vqai=hGuQ8kuc9pgc9s8qqaq=dirpe0xb9q8qiLsFr0=vr0=vr0dc8meaabaqaciaacaGaaeqabaqabeGadaaakeaaiiGacuWFdpWCgaqeamaaBaaaleaacqWFYoGydaWgaaadbaGaem4CamNaemyAaKMaemyBa0gabeaaaSqabaaaaa@34BB@ : 100 * mean of posterior standard deviations

^(3)^ MB : 100 * Mean Bias

^(4)^ sd(*β*_*sim*_) : 100 * standard deviation of posterior means

^(5)^ RMSE : 100 * Root Mean Square Error

^(6)^*π*(*β*) : Coverage proportion *β *∈ *CI*(*β*)

^(7) ^1 - *π*(0) : Non-Coverage proportion 0 ∉ *CI*(*β*)

^(*)^ : Indicator of p-value < 5% from McNemar's test for comparing proportions 1 - *π*(0) under *M*_0 _and *BYM *models.

For E¯h
 MathType@MTEF@5@5@+=feaafiart1ev1aaatCvAUfKttLearuWrP9MDH5MBPbIqV92AaeXatLxBI9gBaebbnrfifHhDYfgasaacH8akY=wiFfYdH8Gipec8Eeeu0xXdbba9frFj0=OqFfea0dXdd9vqai=hGuQ8kuc9pgc9s8qqaq=dirpe0xb9q8qiLsFr0=vr0=vr0dc8meaabaqaciaacaGaaeqabaqabeGadaaakeaacuWGfbqrgaqeamaaBaaaleaacqWGObaAaeqaaaaa@2F5C@ = 46.6 (and *ρ*_*xx *_= 0.95), the mean bias and RMSE were smaller than in the scenario in which E¯h
 MathType@MTEF@5@5@+=feaafiart1ev1aaatCvAUfKttLearuWrP9MDH5MBPbIqV92AaeXatLxBI9gBaebbnrfifHhDYfgasaacH8akY=wiFfYdH8Gipec8Eeeu0xXdbba9frFj0=OqFfea0dXdd9vqai=hGuQ8kuc9pgc9s8qqaq=dirpe0xb9q8qiLsFr0=vr0=vr0dc8meaabaqaciaacaGaaeqabaqabeGadaaakeaacuWGfbqrgaqeamaaBaaaleaacqWGObaAaeqaaaaa@2F5C@ = 23.3. The *β *coverage rate was greater than 94.5% for both models and the proportion was higher for the BYM model. The non-coverage proportions were significantly different, in favor of the *M*_0 _model, for *β *= 0.12. For *β *= 0, the non-coverage proportion was again smaller with the BYM model.

For E¯h
 MathType@MTEF@5@5@+=feaafiart1ev1aaatCvAUfKttLearuWrP9MDH5MBPbIqV92AaeXatLxBI9gBaebbnrfifHhDYfgasaacH8akY=wiFfYdH8Gipec8Eeeu0xXdbba9frFj0=OqFfea0dXdd9vqai=hGuQ8kuc9pgc9s8qqaq=dirpe0xb9q8qiLsFr0=vr0=vr0dc8meaabaqaciaacaGaaeqabaqabeGadaaakeaacuWGfbqrgaqeamaaBaaaleaacqWGObaAaeqaaaaa@2F5C@ = 2.33, the bias increased (up to 6%) and the RMSE was the highest observed in the various cases (14% approx.). The coverage proportion was smaller than in the previous scenario but greater than 92%. The coverage proportion *π*(*β*) was higher with the BYM model than with the *M*_0 _model. While the non-coverage proportions of 0 were close to 1 (except for *β *= 0), the proportions decreased by 16% for *M*_0 _and 13% for BYM for *β *= 0.12. Moreover the non-coverage proportions of 0 for the two models were significantly different and in favor of the *M*_0 _model for *β *= 0.12, 0.21, 0.33.

High autocorrelations thus appear to influence the *over-fitting *effect of the BYM model. The expected disease counts, also modulates the overall accuracy of the estimation. In fact, with highly correlated spatial structure and low disease counts, the bias of the *β *estimate generated by the BYM increases. But, even with a highly autocorrelated covariate and adequate disease counts, when *E*_*i *_is doubled, the BYM model estimates the ecological link with little bias.

## 4 Discussion

A simulation study was conducted in order to assess estimation performance with respect to the ecological association between covariates and health indicators. Key parameters, such as the ecological link, expected disease counts and autocorrelation strength were selected to ensure that the simulation covered realistic situations. The choice of parameters enabled coverage of balanced and unbalanced between- and within-area variabilities.

For moderate autocorrelation structures, both the Poisson model and the BYM model performed well and the estimation performances were similar. Underestimation of ecological links was only observed for high autocorrelations. Overall, the posterior standard deviation of *β *was slightly over-estimated with the BYM model, resulting in conservative results when the true value of *β *was null.

The expected disease counts are also of interest because, with a high autocorrelation, the underestimation of the BYM model is present. In practice, this worst-case scenario can nonetheless be found. Except for the extreme scenario, strong spatial structure and low disease counts, both models perform well, even with strong spatial structure. As a consequence, the BYM model can be used to estimate ecological associations without fearing underestimation. The simulation results show that models accounting for structured and unstructured residuals do not underestimate materially the ecological association. The rational is the following: not accounting for an actual spatial variability leads to strong bias. Thus from a practical point of view, the BYM model should be preferred to the Poisson if spatial autocorrelation of covariate is suspected. Moreover, autocorrelation structure will be first investigated via Moran's I test [15].
